# Identifying Prognostic Significance of RCL1 and Four-Gene Signature as Novel Potential Biomarkers in HCC Patients

**DOI:** 10.1155/2021/5574150

**Published:** 2021-06-28

**Authors:** Jun Liu, Shan-Qiang Zhang, Jing Chen, Zhi-Bin Li, Jia-Xi Chen, Qi-Qi Lu, Yu-Shuai Han, Wenjie Dai, Chongwei Xie, Ji-Cheng Li

**Affiliations:** ^1^Medical Research Center, The Affiliated Yue Bei People's Hospital, Shantou University Medical College, Shaoguan 512025, China; ^2^Institute of Cell Biology, Zhejiang University, Hangzhou 310058, China

## Abstract

**Background:**

Hepatocellular carcinoma (HCC) is a highly malignant disease, and it is characterized by rapid progression and low five-year survival rate. At present, there are no effective methods for monitoring the treatment and prognosis of HCC.

**Methods:**

The transcriptome and gene expression profiles of HCC were obtained from the Cancer Genome Atlas (TCGA) program, International Cancer Genome Consortium (ICGC), and Gene Expression Omnibus (GEO) databases. The random forest method was applied to construct a four-gene prognostic model based on RNA terminal phosphate cyclase like 1 (RCL1) expression. The Kaplan-Meier method was performed to evaluate the prognostic value of RCL1, long noncoding RNAs (AC079061, AL354872, and LINC01093), and four-gene signature (*SPP1, MYBL2, TRNP1*, and *FTCD*). We examined the relationship between RCL1 expression and immune cells infiltration, tumor mutation burden (TMB), and microsatellite instability (MSI).

**Results:**

The results of multiple databases indicated that the aberrant expression of RCL1 was associated with clinical outcome, immune cells infiltration, TMB, and MSI in HCC patients. Meanwhile, we found that long noncoding RNAs (AC079061, AL354872, and LINC01093) and RCL1 were significantly coexpressed in HCC patients. We also confirmed that the four-gene signature was an independent prognostic factor for HCC patients. Ferroptosis potential index, immune checkpoint molecules, and clinical feature were found to have obvious correlations with risk score. The area under the receiver operating characteristic curve values for the model were 0.7–0.8 in the training set and the validation set, suggesting high robustness of the four-gene signature. We then built a nomogram for facilitating the use in clinical practice.

**Conclusion:**

Our study demonstrated that RCL1 and a novel four-gene signature can be used as prognostic biomarkers for predicting clinical outcome in HCC patients; and this model may assist in individualized treatment monitoring of HCC patients in clinical practice.

## 1. Introduction

Hepatocellular carcinoma (HCC) is the most common primary liver cancer worldwide and the third most common cause of cancer-related deaths [1]. Although various diagnostic and treatment strategies, including cascade screening and immunotherapy, have been adopted, the overall incidence and mortality rates of HCC have increased [2]. In Europe and America, the 5-year survival rate for HCC is 18%, and its mortality is second only to pancreatic cancer [3]. Notably, the overall 5-year survival rate for HCC patients in China is only 12% [4]. However, the molecular mechanisms underlying the development of HCC remain unknown. Therefore, identification of new biomarkers that can accurately predict HCC prognosis would be of great significance for monitoring and developing individualized treatment strategies and preventing recurrence of HCC.

RNA terminal phosphate cyclase Like 1 (RCL1) encodes a highly conserved RNA 3-terminal phosphorylation cyclase like protein, which plays a central role in 18s RNA maturation and ribosome biogenesis [5, 6]. RCL1 is widely expressed in various tissues with relatively higher expression in the liver and fat tissues (https://gtexportal.org/home/gene/RCL1). Recent studies showed that RCL1 is expressed in the human brain, and its deletion is associated with the occurrence of depression [7]. Further researches demonstrated that the expression of RCL1 increases with the development of brain, and RCL1 deletion can cause a variety of neurological diseases [8]. However, the expression of RCL1 and its prognostic value in HCC have not been reported.

In this study, the mRNA expression level of RCL1 in HCC tissues and adjacent normal tissues was detected in multiple cohorts, including the TCGA, ICGC, and GES14520 cohorts. Meanwhile, we also analyzed the relationship between RCL1 abnormal expression and poor prognosis of HCC. Furthermore, we explored the potential function of RCL1 in the occurrence of HCC and identified the long noncoding RNAs (LncRNAs) that can be involved in the regulation of RCL1 expression. Finally, we integrated multiple datasets to construct a prediction model of four genes. Thus, we identified that the abnormal expression of RCL1 is associated with poor prognosis of HCC, and RCL1 can used as a potential biomarker for HCC prognosis.

## 2. Materials and Methods

### 2.1. Data Collection and Preprocessing

The TCGA level 3 data for HCC RNA-seq and single nucleotide polymorphism were collected from the UCSC Cancer Genomics Browser (https://genome-cancer.ucsc.edu). Meanwhile, the HCC RNA-seq data (LIRI-JP) and the microarray data (GSE14520, GPL3921) were downloaded from the International Cancer Genome Consortium (ICGC) and Gene Expression Omnibus (GEO), respectively. The RNA-seq of HCC from TCGA and ICGC was performed by log2(FPKM+1), and GSE14520 expression profile data were background-corrected and quantile-normalized using the “limma” package in R, and the “sva” package in R was used to remove batch effects. Corresponding detailed clinical data for HCC patients were also obtained.

### 2.2. Analysis of Expression and Clinical Characteristics

The Wilcoxon test and paired *t*-test were performed to identify the difference in RCL1 expression between HCC tissues and adjacent normal tissues. The Wilcoxon test was also used to check the RCL1 expression in different clinical features. The Kaplan-Meier (K-M) curve was used to explore the effects of RCL1 aberrant expression on HCC clinical outcome. The “surv-cutpoint” function in the survminer R package was used to identify the best split. HCC patients were divided into the high RCL1 expression group and the low RCL1 expression group.

### 2.3. The Association between RCL1 Expression and Immune Cells Infiltration, Tumor Mutation Burden, and Microsatellite Instability

Tumor IMmune Estimation Resource (TIMER) is a tool for estimating immune cells infiltration abundance based on RNA-seq [9]. The TIMER database was used to assess the relationship between RCL1 expression and six immune cell types, including B cells, CD4+ T cells, CD8+ T cells, macrophages, neutrophils, and dendritic cells. The prognostic value of Microsatellite Instability (MSI) and Tumor Mutation Burden (TMB) in HCC was investigated using the K-M plotter based on best separation methods. Subsequently, the correlation between RCL1 aberrant expression and immune cells infiltration/TMB/MSI was evaluated.

### 2.4. Integrated Network and Gene Set Enrichment Analysis (GSEA)

GeneMANIA (http://genemania.org/) was used to construct the protein-protein interaction network of RCL1 and to predict the potential function of RCL1. The network was built through physical interactions, gene coexpression, website prediction, and gene colocalization in GeneMANIA database. The GSEA software (http://www.broadinstitute.org/gsea) was used for pathway enrichment analysis. For Gene sets, a false discovery rate (FDR) was <0.05 and a family‐wise error rate was <0.05.

### 2.5. LncRNAs Coexpression Analysis

Spearman correlation coefficient was used to discriminate the LncRNAs coexpressed with RCL1; and for the LncRNAs with coefficient >0.5, *p* < 0.001 was selected. Then the K-M analysis was performed to estimate the prognostic value of LncRNAs in HCC.

### 2.6. Construction of Risk Signature

The “limma” R package was used to identify the differentially expressed genes between the low RCL1 expression group and the high RCL1 expression group with |log2 fold change FC*|* > 1 and false discovery rate (FDR) < 0.05 as a screening criterion. Gene Ontology (GO) and Kyoto Encyclopedia of Genes and Genomes (KEGG) enrichment analysis for the differentially expressed genes were conducted and visualized using the “clusterProfiler” and “ggplot2” R package. The univariate Cox regression analysis was used to identify genes associated with overall survival (*p* < 0.05). Then the importance of the survival related differential genes was calculated and ranked using the random forest via the “randomForestSRC” R package. Next, the analysis of survival related genes was performed to build a risk signature as follows: risk score = *β*_1_*x*_1_ +*β*_2_*x*_2_+*β*_3_*x*_3_+,…,+ *β*_*n*_*x*_*n*_.

Furthermore, the final gene model was screened by combining the gene number and log-rank *p* value based on the K-M analysis. Univariate and multivariate Cox regression analyses were performed to assess whether the risk signature was an independent prognostic factor. The receiver operating characteristic (ROC) curve was used to evaluate the predictive power of the signature.

### 2.7. Correlation Analyses between Risk Model and Tumor Microenvironment

The immune checkpoint molecules, including PDCD1, CTLA4, CD80, CD86, CD274, PDCD1LG2, CD276, and VTCN1, were identified and differentially expressed in high-risk and low-risk groups. The hypoxia-related genes and the ferroptosis potential index (FPI) were obtained from previous literature and the expression levels in high-risk and low-risk groups were checked [10, 11]. Meanwhile, the association between risk score and survival status, clinical stage, and pathological grade was analyzed.

### 2.8. Construction and Evaluation of the Nomogram

In this study, clinical stage and risk score both were independent prognostic factors for HCC patients. In order to facilitate the potential clinical utility, the nomogram included risk score and clinical stage was constructed for assessing the 1-year, 3-year, and 5-year survival probabilities. Meanwhile the calibration curve of the nomogram evaluated the agreement between the observed rates and predicted overall survival. The ROC and decision curve analysis (DCA) were performed to assess the clinical application quality of the nomogram.

## 3. Results

### 3.1. Clinical Characteristics of the Study Population

In this study, 221, 370, and 232 HCC tissue samples were obtained with corresponding clinical data from the GEO, TCGA, and ICGC databases, respectively. The details of clinical stage, pathological grade, sex, age, and survival rate are shown in Table 1.

### 3.2. Differential Expression Analysis

**Figure 1:**
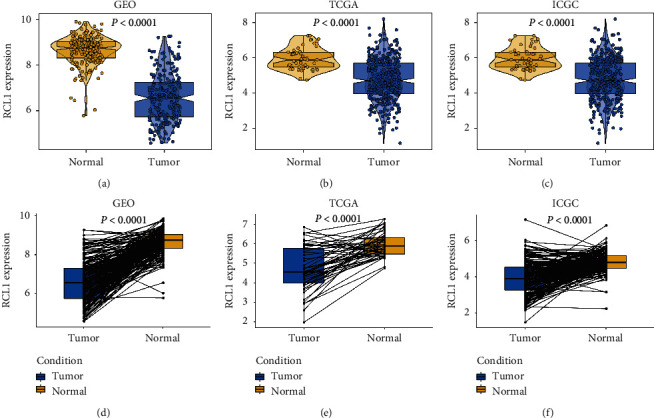
Expression of RCL1 in HCC. (a) The mRNA expression of RCL1 in HCC intratumoral tissue and adjacent tissue based on GEO (A), TCGA (b), and ICGC (c) cohorts. The Wilcoxon test was used to calculate *p* value. Expression distribution of RCL1 in pairwise comparisons in GEO (d), TCGA (e), and ICGC (f) cohorts. Paired *t*-test was performed.

**Figure 2:**
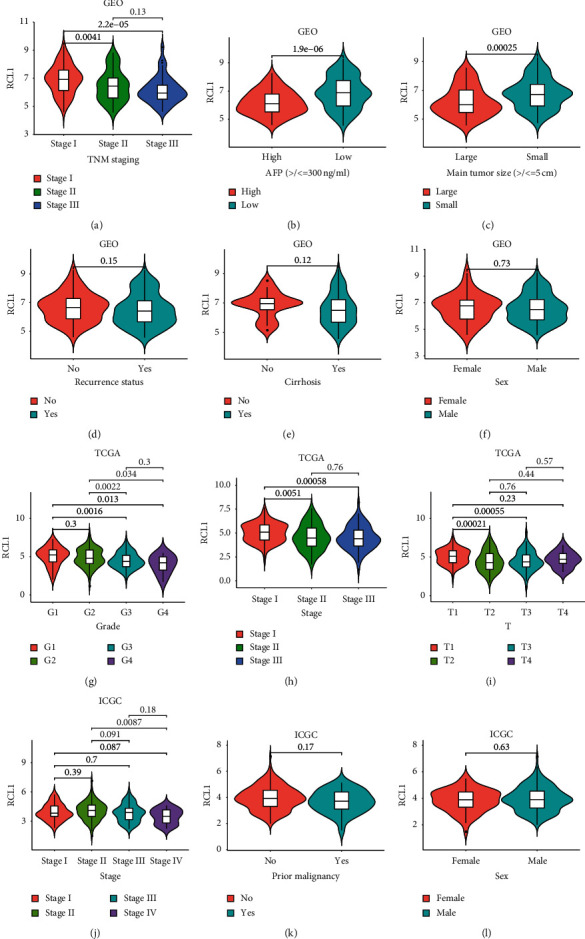
Correlation analysis between RCL1 expression and clinical characteristics. Expression distribution of RCL1 in different TNM stage (a), AFP group (b), tumor size (c), recur status (d), cirrhosis status (e), and sex (f) based on GEO cohort. The expression of RCL1 in different clinicopathologic grade (g), TNM stage (h), and T stage (i) based on TCGA cohort. RCL1 expression and TNM stage (j), prior malignancy (k), and sex (l) correlation in ICGC cohort.

We evaluated the mRNA expression level of RCL1 in HCC intratumoral tissue (IT) and peritumoral normal tissue (PT) based on the GEO, TCGA, and ICGC databases. The results indicated relatively lower RCL1 expression levels in HCC IT, compared with the PT (Figures 1(a)–1(c)). Meanwhile, we further analyzed the RCL1 expression level via paired *t*-test, and the results also indicated lower RCL1 expression levels in HCC IT, compared with the PT (Figures 1(d)–1(f)). Next, we examined the RCL1 expression level in various normal tissues based on Genotype-Tissue Expression (GTEx) data via the HCCDB database (http://lifeome.net/database/hccdb/home.html) [12], and the results showed higher expression levels of RCL1 in the liver tissue (Figure S1(a)). Meanwhile, we also explored the RCL1 expression level in TCGA by the HCCDB database, and the results showed that the difference in RCLI expression between HCC adjacent normal tissue and tumor tissue was highly significant compared with the other cancers (Figure S1(b)). These results suggested that RCL1 was specifically underexpressed in the IT of HCC patients. Moreover, based on the GEO database, it was observed that the RCL1 expression was lower in high pathological stage, high AFP level, and large tumor size compared with the corresponding low pathological stage, low AFP level, and small tumor size (Figures 2(a)–2(f)). The results from the TCGA and ICGC databases were consistent with the results of GEO database (Figures 2(g)–2(l)).

### 3.3. Survival Analysis

**Figure 3:**
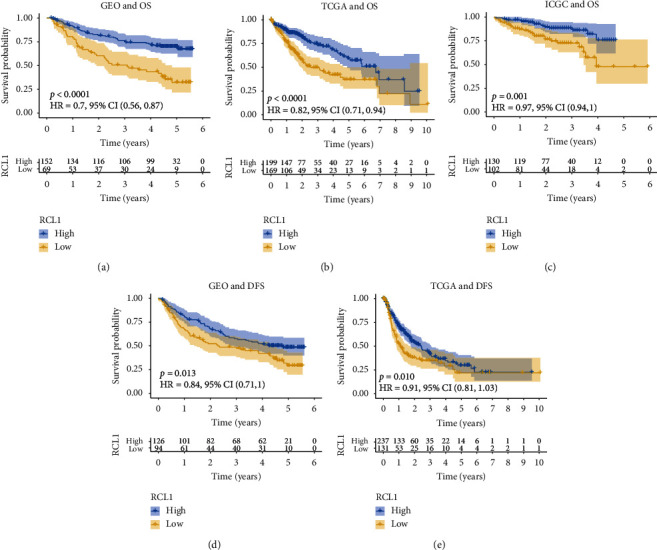
Overall survival (OS) and disease-free survival (DFS) analysis of RCL1. Kaplan-Meier plot of the correlation between RCL1 aberrant expression and OS in GEO (a), TCGA (b), and ICGC (c) cohorts. Association between RCL1 expression and DFS in GEO (d) and TCGA (e) cohorts.

To determine the predictive value of RCL1 in HCC, we performed overall survival (OS) analysis via the K-M method. The results indicated that lower RCL1 expression was associated with poor OS in the TCGA, GEO, and ICGC databases (Figures 3(a)–3(c)). Furthermore, we confirmed that HCC patients with lower RCL1 expression had poor disease-free survival (DFS) in the TCGA and GEO cohorts (Figures 3(d) and 3(e)).

### 3.4. Immune Cells Infiltration, TMB, and MSI Analysis

**Figure 4:**
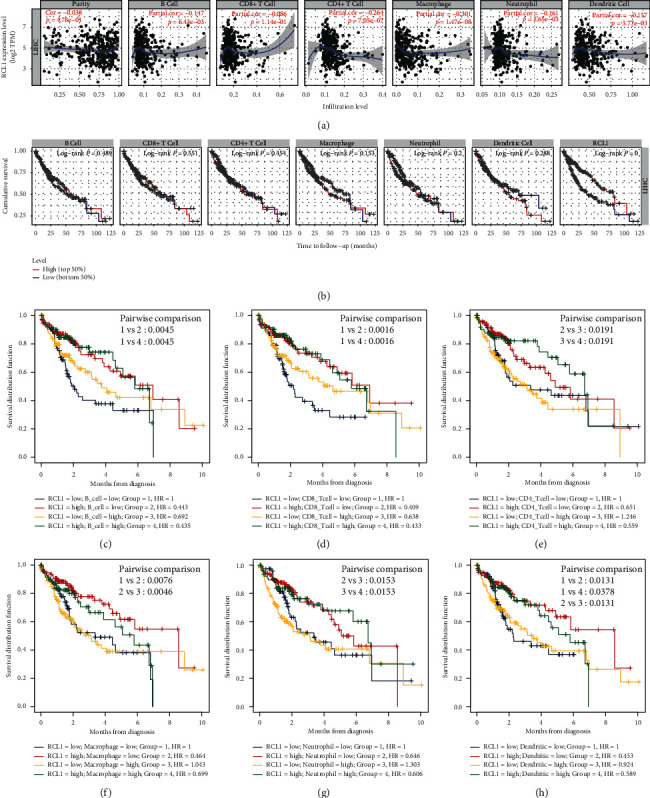
The association between RCL1 expression and immune cell filtration. (a) Assessment of the relationship between RCL1 expression and six-type immune cell score from TIMER database. (b) Overall survival (OS) analysis of six-type immune cell and RCL1 with patients of HCC in TIMER database. OS analysis of combinations of RCL1 expression and B cell (c), CD8+ T cell (d), CD4+ T cell (e), macrophage (f), neutrophil (g), and dendritic cells (h).

We analyzed the relationship between RCL1 expression and immune cells infiltration. The results showed that RCL1 expression was negatively associated with B cells, CD4+ T cells, macrophages, neutrophils, and dendritic cells infiltration score. Particularly, a strong correlation was observed between macrophages and RCLI expression (Figure 4(a)). We further investigated the prognostic value of immune cells infiltration and RCL1 expression in HCC patients based on the TIMER database. The results indicated that immune cells infiltration did not affect OS, while the RCL1 aberrant expression had a significant effect on OS in HCC patients (Figure 4(b)). Furthermore, we divided HCC patients from the TCGA cohort into four groups according to the RCL1 expression and immune cells infiltration score. We found that the lower RCL1 expression level combined with the lower B cells, CD8+ T cells, and dendritic cells infiltration was associated with a poor OS in HCC patients, compared with the higher RCL1 expression level (Figures 4(c), 4(d), and 4(h)). However, the lower RCL1 expression level indicated poor OS, compared with the higher RCL1 expression level in HCC patients regardless of CD4+ T cells and neutrophils infiltration (Figures 4(e) and 4(g)). Meanwhile, the lower RCL1 expression level combined with the higher macrophages score predicted an unfavorable OS in HCC patients (Figure 4(f)). This may be due to the fact that M2 macrophages accounted for a greater ratio of tumor-associated macrophages in HCC tumor microenvironment.

**Figure 5:**
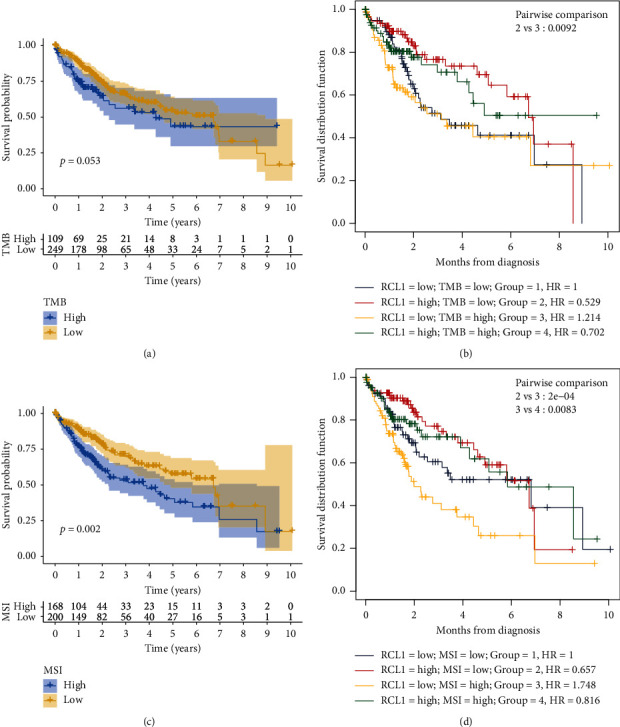
Overall survival (OS) analysis of Microsatellite Instability (MSI) and Tumor Mutation Burden (TMB). (a) The prognostic value of TMB in HCC based on TCGA cohort. (b) OS analysis of combinations of TMB and RCL1 expression in HCC. (c) The prognostic value of MSI in HCC based on TCGA cohort. (d) OS analysis of combinations of MSI and RCL1 expression in HCC.

Previous studies have demonstrated that TMB and MSI are poor prognostic indicators in multiple cancers [13, 14]. In the present study, we found that higher TMB had a tendency for shorter OS, although it was not statistically significant (Figure 5(a)). Meanwhile, HCC patients with the lower RCL1 expression level and higher TBM had unfavorable OS, and the patients with lower TMB and higher RCL1 expression had better OS (Figure 5(b)). Subsequently, we analyzed the potential role of MSI in HCC, and the results demonstrated that higher MSI was associated with poor OS (Figure 5(c)). The patients with lower RCL1 expression and higher MSI had worst OS among the four groups (Figure 5(d)).

### 3.5. Enrichment Analysis

**Figure 6:**
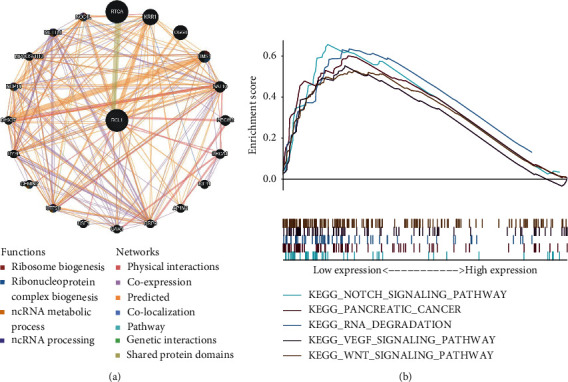
Protein-protein interaction network (PPI) and Gene Set Enrichment Analysis (GSEA). (a) The GeneMANIA online tool was performed to construct the PPI of RCL1. (b) GSEA of RCL1 using GSEA software.

To understand the potential function of RCL1 in HCC development, the protein-protein interaction network was constructed through GeneMANIA database. The results exhibited that RCL1 may interact with RTCA, KRR1, OGG1, and BMS1, and these genes may be involved in ribosome biogenesis, ribonucleoprotein complex biogenesis, and ncRNA processing (Figure 6(a)). We also found that Notch signaling pathway, pancreatic cancer, RNA degradation, VEGF signaling pathway, and WNT signaling pathway were enriched in the low RCL1 expression group via GSEA (Figure 6(b)).

### 3.6. LncRNAs Coregulated Analysis

**Figure 7:**
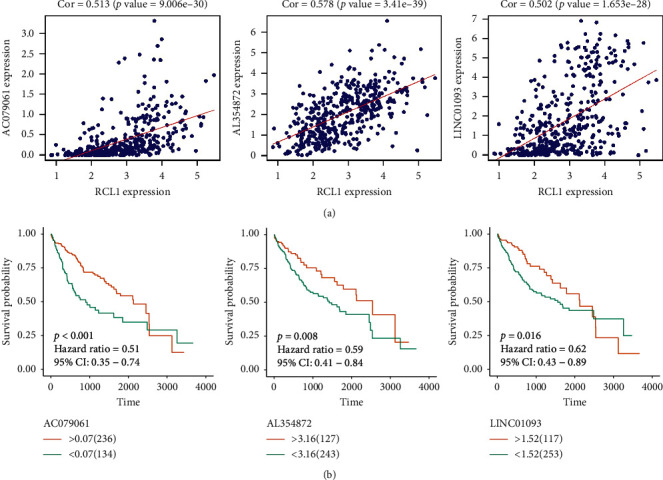
LncRNA analysis of RCL1 coexpression. (a) Identification of LncRNA with RCL1 coexpression coefficients greater than 0.5. (b) The overall survival of AC079061, AL354872, and LINC01093 in HCC patients.

LncRNAs are known to be involved in various stages of tumorigenesis. In this study, we explored whether LncRNAs were involved in regulating the expression of RCL1 through coexpression analysis. Based on the TCGA cohort, we selected the LncRNAs (AC079061, AL354872, and LINC01093) with correlation coefficients more than 0.5 and RCL1 as potential regulatory genes (Figure 7(a)). The K-M survival analysis also showed that the aberrant expression of these LncRNAs was associated with poor clinical outcome (Figure 7(b)).

### 3.7. Differential Genes and Enrichment Analysis

**Figure 8:**
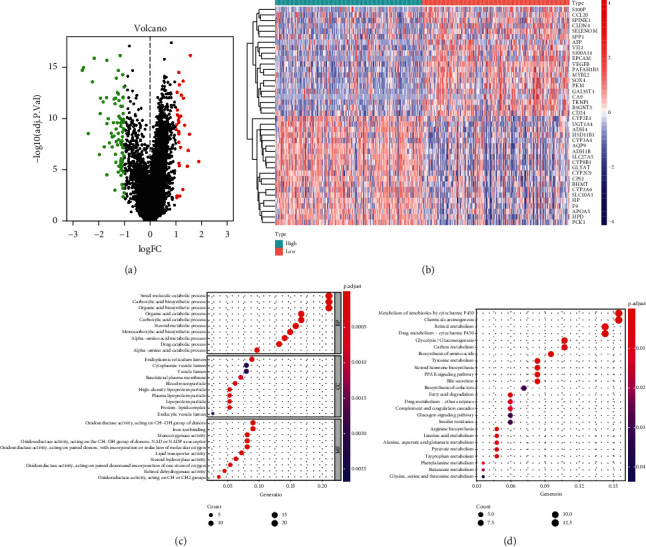
Differential expression genes (DEGs) and enrichment analysis. (a) The volcano plot of DEGs between RCL1 low expression and high low expression group. (b) Heatmap of top 40 DEGs. Gene Ontology (GO) enrichment (c) and Kyoto Encyclopedia of Genes and Genomes (KEGG) enrichment (d) analysis of DEGs.

We split HCC patients from the TCGA cohort into two groups based on RCL1 expression, and the differentially expressed genes were identified. The volcano plot showed 30 upregulated and 90 downregulated genes (Figure 8(a)). The expression of top 20 upregulated and downregulated genes is shown in the heatmap (Figure 8(b)). Moreover, we performed GO and KEGG pathway enrichment analysis for differentially expressed genes (Figures 8(c) and 8(d)); and the results indicated that differentially expressed genes were mainly involved in multiple metabolic processes.

### 3.8. Construction and Verification of Four-Gene Signature

**Figure 9:**
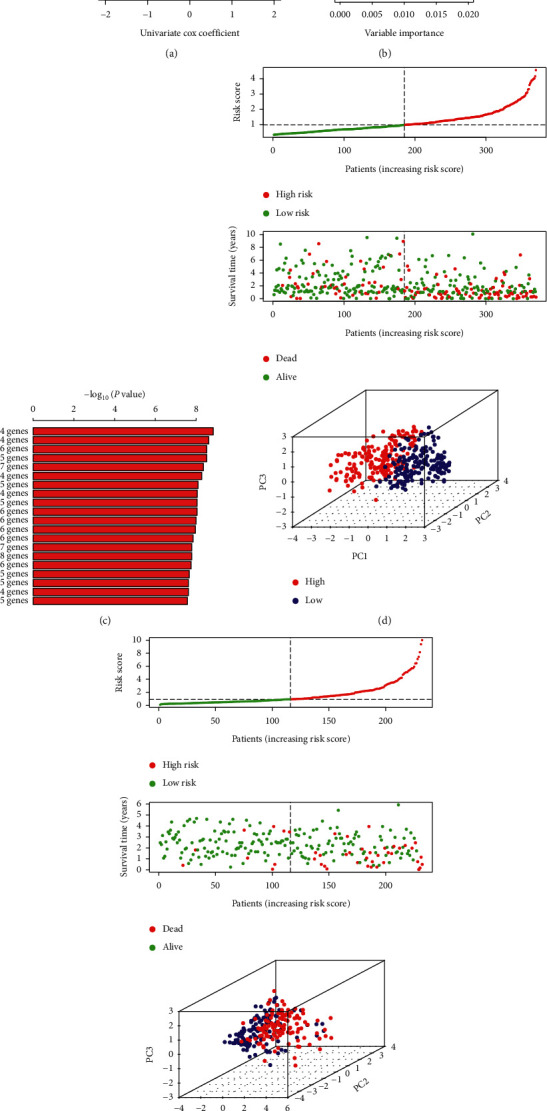
Screen of important genes and construction of gene signature. (a) Volcano plot displayed the survival-related genes through univariate Cox regression analysis. (b) Identification of relative important genes via random survival forest algorithms. (c) Top 20 gene signatures were ranked by –log 10 *p* value using Kaplan-Meier plotter calculated. The distribution of risk score and survival time in high-risk and low-risk groups in training (d) and validation (e) sets. Principal Component Analysis (PCA) showed the difference between high-risk and low-risk groups.

In the TCGA cohort, we used the univariate Cox regression analysis to select the OS related differential genes (*p* < 0.05) and found that 43 genes were associated with OS (Figure 9(a)). We next ranked the relative importance of 43 OS related genes by the random forest, and top 10 genes were selected (SPP1, CDC20, MYBL2, UBE2C, TRNP1, MTTP, CDO1, CYP2C9, FTCD, and FMO3, Figure 9(b)). Based on these 10 genes, more than 1000 risk models were constituted. Subsequently, we further screened the risk model with smallest *p* value based on the K-M plotter (Figure 9(c)); and the four-gene signature was then built as follows: risk score = 0.085*∗*SPP1 + 0.125*∗*MYBL2 + (–0.113*∗*FTCD) + 0.091*∗*TRNP1. The training set (TCGA cohort) and the validation set (ICGC cohort) were divided into the high-risk group and the low-risk group based on the median value of risk score. We found that the patients in the high-risk group had higher risk score and poor survival status compared with the low-risk group in the training set and the validation set (Figures 9(d) and 9(e)). Also, Principal Component Analysis (PCA) results exhibited a good separation between the high-risk group and the low-risk group (Figures 9(d) and 9(e)).

**Figure 10:**
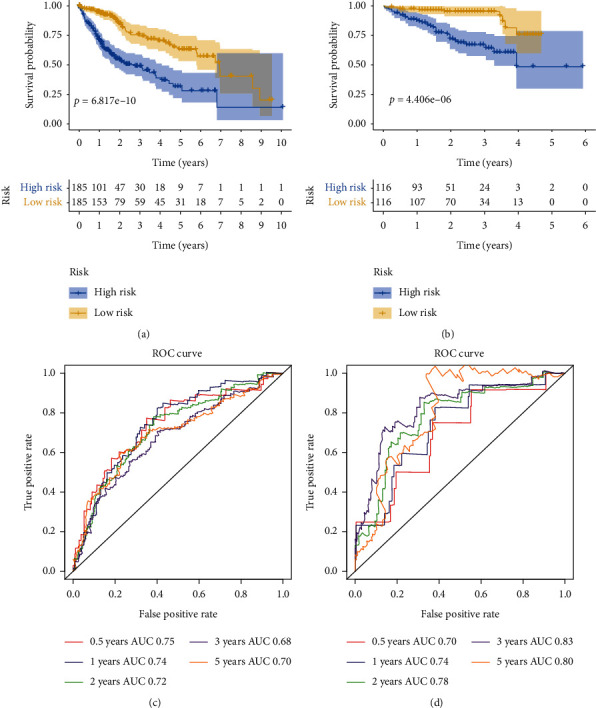
Overall survival (OS) and receiver operating curve (ROC) analysis of four-gene signature. (a) The OS analysis of four-gene signature in TCGA cohort. (b) The OS analysis of four-gene signature in ICGC cohort. (c) The ROC of four-gene signature for 0.5, 1, 2, 3, and 5 years in TCGA cohort. (d) The ROC of four-gene signature for 0.5, 1, 2, 3, and 5 years in ICGC cohort.

Subsequently, the K-M plotter was performed to evaluate the OS difference between the high-risk group and the low-risk group. The results indicated that patients in the high-risk group had significantly poorer OS than that of patients in the low-risk group in the TCGA and ICGC cohorts (Figures 10(a) and 10(b)). Meanwhile, the area under the ROC curve (AUC) was applied to assess the predictive power of four-gene signature. The AUC for 0.5, 1, 2, 3, and 5 years were 0.75, 0.74, 0.72, 0.68, and 0.70, respectively, in the TCGA cohort (Figure 10(c)); and the AUC for 0.5, 1, 2, 3, and 5 years were 0.70, 0.74, 0.78, 0.83, and 0.80, respectively, in the ICGC cohort (Figure 10(d)). These results demonstrated excellent prediction ability of the four-gene signature for OS in HCC patients.

**Figure 11:**
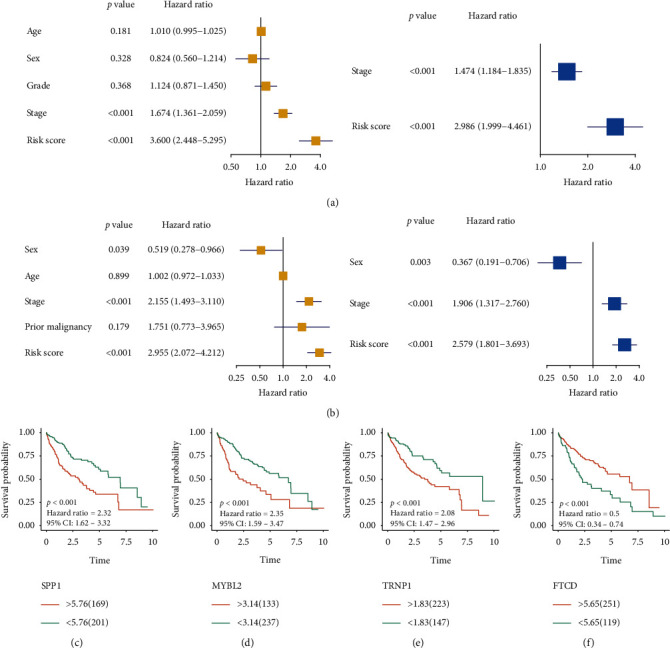
Independent prognostic value of the four-gene signature and overall survival (OS) analysis of four genes. Univariate and multivariate COX regression analysis of the four-gene signature in TCGA (a) and ICGC (b) cohorts. Kaplan-Meier plotter analysis of SPP1 (c), MYBL2 (d), TRNP1 (e), and FTCD (f) in TCGA cohort.

Furthermore, univariate and multivariate Cox regression analyses were carried out to evaluate whether the four-gene signature was a prognosis-related marker. In the TCGA cohort, the results of univariate Cox regression analysis suggested that clinical stage and risk score were associated with OS in HCC patients (Figure 11(a)). The multivariate Cox regression analysis confirmed that clinical stage and risk score were independent prognostic factors (Figure 11(a)). We further verified this in the ICGC cohort, and the results showed that clinical stage and risk score remained independent OS predictor (Figure 11(b)). Finally, we evaluated the prognostic value of SPP1, MYBL2, TRNP1, and FTCD genes via the K-M method in the TCGA cohort and found that these genes were associated with OS (Figures 11(c)–11(f)).

### 3.9. Association Risk Score with Immune Checkpoint, Hypoxia, FPI, and Clinical Feature

**Figure 12:**
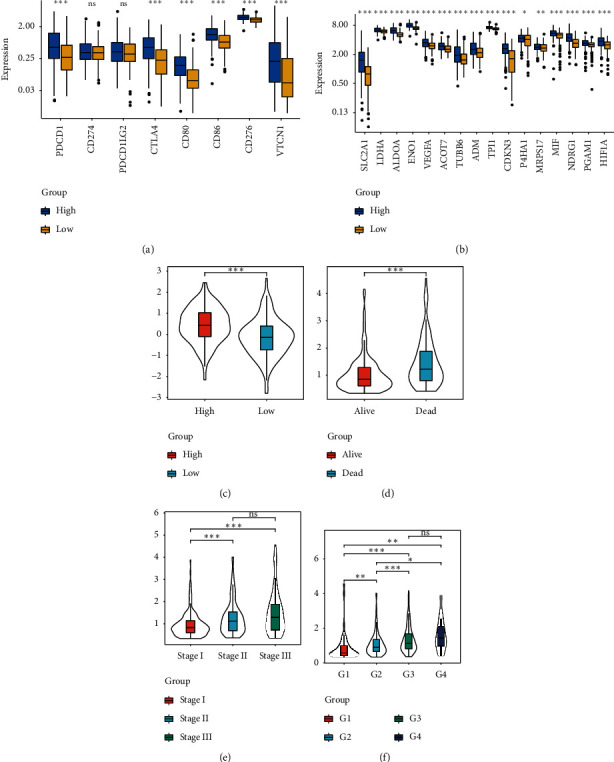
Association analyses of risk score and immune checkpoint molecules (a), hypoxia-related genes (b), ferroptosis potential index (c), survival status (d), clinical stage (e), and pathological stage (f).

The expression of immune checkpoint molecules was strongly associated with immune escape. We analyzed the expression of eight common immune checkpoint molecules between high-risk and low-risk groups based on TCGA cohort. The results indicated that PDCD1, CTLA4, CD80, CD86, CD276, and VTCN1 presented higher expression in high-risk group compared to low-risk group (Figure 12(a)). Meanwhile, the expressions of sixteen hypoxia-related genes were also higher in the high-risk group compared with the low-risk group (Figure 12(b)). These results suggested that patients with high risk showed unfavorable immune microenvironment. Ferroptosis is mediated by iron metabolism, a novel type of cell death, and playing crucial role in prompting aggressive malignancies [15]. In this study, we found that the patients with high risk had higher FPI than those with low risk (Figure 12(c)). Furthermore, we explored the relationship between risk score and clinical feature. The results demonstrated that risk-score was remarkably increased in dead patients compared with living patients (Figure 12(d)). Meanwhile, the risk-score presented an elevated tendency with advanced pathological stage and grade (Figures 12(e) and 12(f)).

### 3.10. Constructing and Evaluating a Nomogram

**Figure 13:**
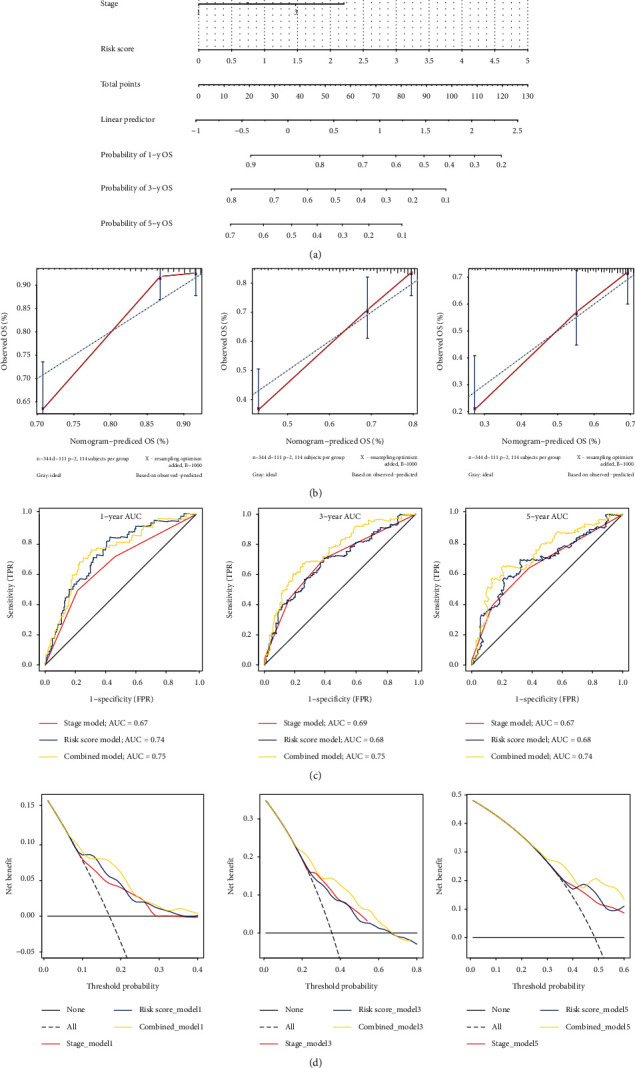
Constructing and verifying a nomogram. (a) The nomogram was built combining the four-gene signature and clinical stage; the total point was calculated by adding up the stage and risk score. (b) The calibration curve of overall survival in TCGA cohort at 1, 3, and 5 years. *X*-axis is the result of the nomogram; *Y*-axis is the result of the observed OS. (c) AUC of the stage model, risk-score model, and nomogram on 1-year, 3-year, and 5-year overall survival prediction in TCGA cohort. (d) *Y*-axis represents net benefits, and *X*-axis shows the threshold probabilities of 1, 3, and 5 years. The grey broken line shows the assumption that no patients have 1-year, 3-year, and 5-year overall survival.

A nomogram was constructed incorporating two independent prognostic factors, risk score, and clinical stage to facilitate clinical application for predicting the probability of 1-year, 3-year, and 5-year overall survival in the TCGA cohrt (Figure 13(a)). Next, the calibration cure was performed to assess the classification performance of nomogram, and the diagonal dashed line showed the best prediction. The calibration cure suggested that the nomogram had a good predictive ability (Figure 13(b)). Meanwhile, the ROC of the nomogram was the largest, and the AUC of 1, 3, and 5 years were above 0.7 (Figure 13(c)). The DCA also showed that the nomogram had the best net benefit compared to the risk model and stage model (Figure 13(d)).

## 4. Discussion

RCL1, an RNA 3′-terminal phosphate cyclase-like protein, is ubiquitously expressed in various tissues and has been shown to participate in the ribosome biogenesis, rRNA processing, and Gene Expression pathway (https://www.genecards.org/cgi-bin/carddisp.pl?gene=RCL1) [6, 16–18]. Defects in the ribosomal biogenesis can result in cirrhosis and P53 activation and are associated with tumorigenesis [19, 20]. The results from the GETx database showed that RCL1 was most abundantly expressed in the liver compared with the other normal tissues. Meanwhile, we found that RCL1 was downregulated in HCC tissue, and lower expression of RCL1 was associated with poor OS, based on the GEO, TCGA, and ICGC databases.

The tumor microenvironment (TME) is crucial for tumor initiation and worsening [21, 22]. Immune cells are important components of TME; in particular, tumor associated macrophages (TAM) are the most abundant cells in TME [23, 24]. Moreover, previous studies have found that gene expression was associated with immune cell infiltration and affected the prognosis of oncology patients [25, 26]. In this study, we analyzed the relationship between RCL1 expression and immune cells infiltration, and the results indicated that the expression level of RCL1 was strongly associated with macrophages infiltration. Meanwhile, the K-M analysis showed that HCC patients with the lower RCL1 expression level and higher macrophages infiltration score had poor OS. Previous studies revealed that the Notch signaling pathway and the WNT signaling pathway are involved in regulating TME [27, 28]. In our study, the GSEA showed that the gene in the low RCL1 expression group was mainly enriched in the Notch signaling pathway and WNT signaling pathway. In addition, we also explored the role of TMB and MSI in HCC and analyzed the correlation of RCL1 aberrant expression associating TMB/MSI with OS. The results indicated that RCL1 aberrant expression was related to TME and genome instability in HCC, and RCL1 could act as a potential prognostic biomarker for HCC.

LncRNAs play a crucial role in regulating gene expression and stability [29, 30]. In this study, three survival-related LncRNAs, AC079061, AL354872, and LINC01093, were identified, and these LncRNAs had a strong correlation with RCL1 expression. LINC01093 has been shown to be significantly downregulated in HCC and was correlated with tumor cell proliferation and metastasis [31–33]. However, the function of AC079061 and AL354872 in HCC remained unclear. This study showed that AC079061, AL354872, and LINC01093 were associated with OS and may interact with RCL1.

Recently, the clinical application of 21-gene signature in breast cancer has been demonstrated [34, 35]. Currently, many studies have focused on constructing risk model for oncology patients in order to evaluate the clinical outcome. Liu et al. and Cheng et al. constructed four-transcription factor model and five pseudogenes for predicting the clinical outcome of glioblastoma multiforme and brain lower-grade glioma, respectively [36, 37]. Liang et al. built a 10-gene signature for predicting OS in HCC patients through the least absolute shrinkage and selection operator (LASSO) method [38]. Du et al. showed that seven-mRNA signature was related to recurrence of HCC [39]. In addition, Li et al. also identified a novel six-gene model as biomarkers for predicting HCC OS [40]. Despite these studies, the expression of mRNA as a biomarker for HCC prognosis and therapeutic monitoring still remains exploratory. In this study, the random forest method was applied to screen the OS-related genes, and the risk model was further selected according to the gene number and log rank *p* value of K-M. Finally, the four-gene signature (SPP1, MYBL2, TRNP1, and FTCD) was identified from more than 1000 combinations and splitting the HCC patients with different OS into the low-risk and high-risk groups in the TCGA and ICGC cohorts. Meanwhile, the ROC analysis showed that the four-gene signature had excellent predictive capacity for OS in HCC patients. Correlation analysis exhibited that the patients with high risk had relatively harsh immune microenvironments and FPI. Furthermore, the risk score was also positively related to clinical stage and grade. In this study, FTCD was identified as a protective factor, whereas SPP1, MYBL2, and TRNP1 were risk factors for HCC patients. Zhang et al. reported FTCD as a factor of six-gene model for predicting OS in HCC patients via single-cell data [41]. Moreover, FTCD has been reported to interact with HIF-1*α* and prompted chemosensitivity in HepG2 cells [42]. Several studies have shown that SPP1 is a poor prognostic factor for HCC, and upregulation of SPP1 could induce HCC cell proliferation [43]. Mybl2 can activate cell cycle related pathways and can enhance HCC cell proliferation and motility. It has been shown that targeting Mybl2 can improve the clinical outcome in HCC patients with mutated P53 [44, 45]. TRNP1 plays an important role in neural development and regulating cell self-renewal [46, 47]. However, the role of TRNP1 in HCC development and prognosis still remains unclear.

Although we analyzed the prognostic value of RCL1 and four-gene signature in multiple datasets, there are still some limitations in this study. Our study explored the relationship between LncRNAs and RCL1, which needs to be further verified *in vitro* and *in vivo*. Moreover, the clinical value of the four-gene signature requires further investigation in a larger cohort.

In conclusion, based on the analysis of 823 HCC transcriptome profiles, our study suggested that RCL1 could serve as a novel potential prognostic biomarker for predicting OS and DFS in HCC patients. Meanwhile, AC079061, AL354872, LINC01093, and four-gene signature can be used to predict the OS in HCC patients.

## Figures and Tables

**Table 1:** The clinical characteristics of HCC patients.

Clinical characteristics	—	TCGA	%	ICGC	%	GEO	%
—	—	370	—	232	—	221	
Survival status	Survival	244	65.95	189	81.47	136	61.5
	Death	126	34.05	43	18.53	85	38.5

Age	≤65 years	232	62.70	90	38.79	200	90.5
	>65 years	138	37.30	142	61.21	21	9.5

Gender	Male	249	67.30	171	73.71	191	86.4
	Female	121	32.70	61	26.29	30	13.6

Histological grade	G1	55	14.86	NA	—	NA	—
	G2	177	47.84	NA	—	NA	—
	G3	121	32.70	NA	—	NA	—
	G4	12	3.24	NA	—	NA	—

Stage	I	171	46.22	36	15.52	93	42.1
	II	85	22.97	106	15.69	77	34.8
	III	85	22.97	71	30.60	50	22.6
	IV	5	1.35	19	8.19	NA	0.5

T classification	T1	181	48.92	NA	—	NA	—
	T2	93	25.14	NA	—	NA	—
	T3	80	21.62	NA	—	NA	—
	T4	13	3.51	NA	—	NA	—
	TX	1	0.27	NA	—	NA	—

M classification	M0	266	71.89	NA	—	NA	—
	M1	4	1.08	NA	—	NA	—
	MX	100	27.03	NA	—	NA	—

N classification	N0	252	68.11	NA	—	NA	—
	N1	4	1.08	NA	—	NA	—
	NX	113	30.54	NA	—	NA	—

Prior malignancy	No	NA	NA	202	87.07	NA	—
	Yes	NA	NA	30	12.93	NA	—

Tumor size	Small	NA	—	NA	—	140	63.3
	Large	NA	—	NA	—	80	36.2

Cirrhosis	No	NA	—	NA	—	18	8.1
	Yes	NA	—	NA	—	203	91.9

## Data Availability

The datasets generated and/or analyzed during the current study are available in the TCGA (https://portal.gdc.cancer.gov/), GEO (https://www.ncbi.nlm.nih.gov/geo/), and ICGC (https://icgc.org/) repository.

## Supplementary Materials

Supplementary Materials Figure S1: the expression of RCL1 in Genotype-Tissue Expression (GTEx) and pan-cancers. (a) The expression level of RCL1 in normal tissues based on the GTEx database. (b) The expression level of RCL1 in pan-cancers based on TCGA database.
