# Supplementary material for: Identifying Prognostic Significance of RCL1 and Four-Gene Signature as Novel Potential Biomarkers in HCC Patients
Source: J Oncol. 2021 Jun 28;2021:5574150. doi: 10.1155/2021/5574150 (PMC8260302; doi:10.1155/2021/5574150)
Supplement: Supplementary Materials — Figure S1: the expression of RCL1 in Genotype-Tissue Expression (GTEx) and pan-cancers. (a) The expression level of RCL1 in normal tissues based on the GTEx database. (b) The expression level of RCL1 in pan-cancers based on TCGA database. [file 5574150.f1.docx]

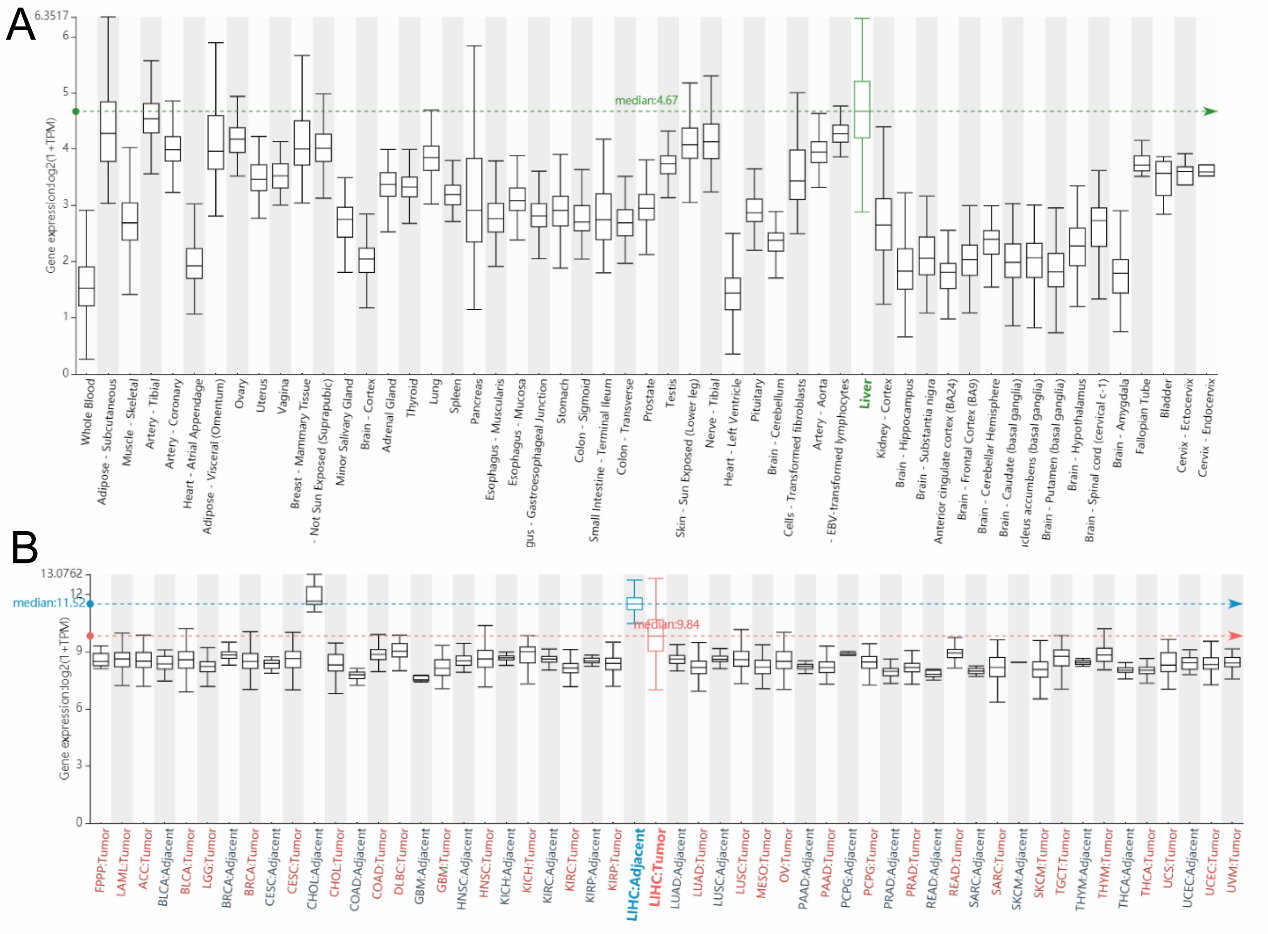


Supplementary Figure 1. The expression of RCL1 in Genotype-Tissue Expression (GTEx) and pan-cancers. A. The expression level of RCL1 in normal tissues based on the GTEx database. B. The expression level of RCL1 in pan-cancers based on TCGA database.
